# Machine learning in general practice: scoping review of administrative task support and automation

**DOI:** 10.1186/s12875-023-01969-y

**Published:** 2023-01-14

**Authors:** Natasha Lee Sørensen, Brian Bemman, Martin Bach Jensen, Thomas B. Moeslund, Janus Laust Thomsen

**Affiliations:** 1 Center for General Practice at, Aalborg University, Aalborg, Denmark; 2 Dept. of Architecture, Design and Media Technology, Aalborg University, Aalborg, Denmark

**Keywords:** General practice, Primary Health Care, Health Services Administration, Organization and Administration, Artificial intelligence, Machine Learning

## Abstract

**Background:**

Artificial intelligence (AI) is increasingly used to support general practice in the early detection of disease and treatment recommendations. However, AI systems aimed at alleviating time-consuming administrative tasks currently appear limited. This scoping review thus aims to summarize the research that has been carried out in methods of machine learning applied to the support and automation of administrative tasks in general practice.

**Methods:**

Databases covering the fields of health care and engineering sciences (*PubMed, Embase, CINAHL with full text, Cochrane Library, Scopus, and IEEE Xplore)* were searched. Screening for eligible studies was completed using Covidence, and data was extracted along nine research-based attributes concerning general practice, administrative tasks, and machine learning. The search and screening processes were completed during the period of April to June 2022.

**Results:**

1439 records were identified and 1158 were screened for eligibility criteria. A total of 12 studies were included. The extracted attributes indicate that most studies concern various scheduling tasks using supervised machine learning methods with relatively low general practitioner (GP) involvement. Importantly, four studies employed the latest available machine learning methods and the data used frequently varied in terms of setting, type, and availability.

**Conclusion:**

The limited field of research developing in the application of machine learning to administrative tasks in general practice indicates that there is a great need and high potential for such methods. However, there is currently a lack of research likely due to the unavailability of open-source data and a prioritization of diagnostic-based tasks. Future research would benefit from open-source data, cutting-edge methods of machine learning, and clearly stated GP involvement, so that improved and replicable scientific research can be done.

**Supplementary Information:**

The online version contains supplementary material available at 10.1186/s12875-023-01969-y.

## Introduction

Patients presenting with any illness that requires medical care will often come in first contact with primary care, which places a significant burden on general practice clinics, facilities, and workers [1]. General practitioners (GPs) must diagnose, monitor, and manage treatment plans, as well as provide preventative medicine and screening – frequently under pressing time constraints due to the need to visit other patients or meet laboratory demands [2]. In between consultations, GPs spend considerable additional time handling referrals, admissions, communications, and other administrative tasks. It is not surprising that such a high volume of patients and the nature of the work involved in providing primary care have been in recent years sources of increasing stress for GPs and potentially diminishing quality of care [3].

Artificial intelligence (AI) has the potential to provide considerable support for various tasks within primary care [4]. Current research in this area has focused primarily on improving decision making during patient care e.g., when identifying undetected diagnoses or classifying existing diseases [5–7]. However, a significant amount of a GP’s time is now spent on handling various administrative tasks that may only be indirectly associated with patient care and have high potential for being fully automated [4]. *Administrative tasks* in general practice may be defined as tasks secondary to providing patient care, typically carried out by either administrative personnel or the GP, that help to support other tasks primarily carried out by the GP in service towards the direct and immediate health of the patient. Willis et al. [8] have identified several such highly automatable administrative tasks in general practice, including “payroll and managing finances, checking and sorting post, printing letters, communicating with patients through texting, management of paper archives (onsite or offsite), transcription, email account management, letter scanning, checking for errors in paperwork and internal communications (e.g., messages to staff or new employee inductions)” (p. 6). Despite this definition and examples, it is not always clear what differentiates an administrative task from other tasks carried out in a primary care setting as general practice clinical duties may differ from country to country, and primary care is constantly evolving.

While numerous research efforts in AI addressing the basis for these administrative tasks outside of general practice exist [9–11], it is not clear the extent to which similar efforts have been made to solve these problems in the context of general practice. A general practice context presents a unique set of challenges concerning e.g., access to data, patient-doctor oriented needs, and cross-disciplinary collaboration, among many others. Unfortunately, most of these methods applied to problems in general practice currently appear focused on those aimed at supporting diagnosis [12] rather than those targeted at administrative tasks. Modern machine learning architectures based on artificial neural networks, however, tend to steadily improve with the increasing size of available data and have a great potential for addressing a variety of administrative tasks in general practice [13].

Due to growing research interests in machine learning methods for general practice, there is a need to evaluate existing literature on the role of such methods in support of the seemingly less prioritized administrative tasks that have been suggested as frequently the most time-consuming for GPs and have the greatest potential for being fully automated. This scoping review will thus provide important knowledge on the current applications, limitations, and issues concerning the future development of machine learning based AI for administrative tasks in general practice.

### Objectives

In this paper, we present a scoping review aimed at providing an overview of the research carried out in machine learning applied to the support and automation of time-consuming administrative tasks in general practice. Importantly, this review characterizes this research along the following three topics: (i) *General practice*, in terms of identifying the broad class of problems to be solved, the kind of data frequently employed, and the role of GPs in the actual research; (ii) *Administrative task*, in terms of defining the specific tasks addressed, the criteria to be improved or made more efficient, and the extent to which these tasks can be automated; and (iii) *Machine learning*, in terms of identifying the machine learning problem used to model the administrative task, the methods employed, and the evaluation measures and results reported.

## Methods

In this section, we detail the methods used in this scoping review, which adhere to the guidelines outlined in *Preferred Reporting Items for Systematic reviews and Meta-Analyses extension for Scoping Reviews (PRISMA-ScR)* [14]. See Additional file 1 for the PRISMA-ScR Checklist.

### Databases

Due to the cross-disciplinary nature of our stated objectives, it is necessary that diverse information sources covering both general practice and the engineering sciences are considered. In order to identify all possibly relevant studies within these domains, the following bibliographic databases were used: *PubMed, Embase, CINAHL with full text, Cochrane Library, Scopus, and IEEE Xplore* – the latter two representing the foremost databases used in the engineering sciences. Prior to searching, unwanted studies were filtered out from each of the information sources according to the following three constraints: (i) publications should be in either peer-reviewed conferences or peer-reviewed journals, (ii) the year of publication should be between 1990 and 2022 (inclusive on either side), and (iii) the written language should be English. The range of publication dates was chosen due to the relatively recent rise of data-driven methods of AI beginning around 1990.

### Eligibility criteria

All studies were required to meet three eligibility criteria concerning their research focus and a further two eligibility criteria concerning the type and availability of the research. The five eligibility criteria were as follows: (i) general practice setting and current problem, (ii) administrative task to be solved, (iii) machine learning method(s) used, (iv) proper study design, and (v) abstract and full text available. Regarding (i), a general practice setting here refers to the stated presence of patient populations having received primary care regardless of the physical location (e.g., general practice clinic vs. primary care outpatient center). Thus, all studies concerning patient populations from secondary care, or any other kind of healthcare were excluded. A current general practice problem refers to the requirement that all included studies must focus on a problem presently found within general practice and not one that might be encountered in the future. Additionally, the data used in all included studies did not need to have been collected from a general practice nor comprise notes from actual GPs. With respect to (ii), an administrative task must be one matching the definition provided in the introduction with possible examples including scheduling, communication, or care planning. Thus, all studies concerning e.g., diagnosis, screening, or treatment of disease were excluded. Additionally, all administrative tasks must be for the benefit of the individual general practice clinic and not for a governing region, municipality, or nation-wide objective. In criterion (iii), all included studies must use machine learning methods (i.e., those beyond classical statistical tests and descriptive statistics), such as artificial neural networks, k-nearest neighbors, and support vector machines, among many others, that belong to either of the major paradigms of supervised, semi-supervised, unsupervised, transfer, federated, or reinforcement learning. Studies that use machine learning solely for data mining purposes with no direct goal oriented towards primary care were excluded e.g., those using machine learning to evaluate the prevalence or characteristics of diagnoses. Regarding (iv), a proper study design was one in which original research, not presented as a review or protocol, was completed. With respect to (v), all publications must have both an abstract and full text available, meaning that abstract-only publications such as extended abstracts were excluded.

### Search process

A comprehensive search of the six databases was made by the first author (NLS) on April 20^th^, 2022, using a set of search strategies drafted by NLS and further refined by the remaining authors based on the authors’ own PICO search (available in Additional file 2) without a comparator and using the aforementioned eligibility criteria. The search terms used were primarily taken from the bibliographic databases’ thesaurus systems and other free-text words relevant to the objectives of this scoping review. Search words corresponding to the pre-defined highly automatable administrative tasks in general practice provided in [8] were initially included in the search strategies, however, they were found to be too narrow for the chosen bibliographic databases and so more general database keywords related to these pre-defined administrative task terms were included instead. Table 1 shows the final search strategy for the PubMed database while the search strategies for the remaining bibliographic databases can be found in Additional file 2.

**Table 1:** Search strategy for the PubMed database consisting of search terms covering general practice, machine learning, and administrative tasks

Number	Search terms
#1	"general practice"[MeSH Terms] OR "general practice"[Title/Abstract] OR "general medicine"[Title/Abstract] OR "primary medical care"[Title/Abstract] OR "primary health care"[MeSH Terms] OR "primary health care"[Title/Abstract] OR "primary healthcare"[Title/Abstract] OR "health care primary"[Title/Abstract] OR "healthcare primary"[Title/Abstract] OR "primary care"[Title/Abstract] OR "family practice"[MeSH Terms] OR "family practice"[Title/Abstract] OR "family medicine"[Title/Abstract] OR "family medicine practice"[Title/Abstract] OR "private practice"[MeSH Terms] OR "private practice"[Title/Abstract] OR "first line care"[Title/Abstract]
#2	"machine learning"[MeSH Terms] OR "machine learning"[Title/Abstract] OR "learning machine"[Title/Abstract] OR "supervised machine learning"[MeSH Terms] OR "supervised learning"[Title/Abstract] OR "supervised machine learning"[Title/Abstract] OR "unsupervised machine learning"[MeSH Terms] OR "unsupervised machine learning"[Title/Abstract] OR "unsupervised learning"[Title/Abstract] OR "reinforcement learning"[Title/Abstract] OR "reinforcement machine learning"[Title/Abstract] OR "semi supervised machine learning"[Title/Abstract] OR "semi supervised learning"[Title/Abstract] OR "deep learning"[MeSH Terms] OR "deep learning"[Title/Abstract] OR "transfer learning"[Title/Abstract] OR "federated learning"[Title/Abstract]
#3	"administration and organization"[Title/Abstract] OR "administration and planning"[Title/Abstract] OR "management"[Title/Abstract] OR "management information systems"[MeSH Terms] OR "organization and administration"[MeSH Terms] OR "organization and administration"[Title/Abstract] OR "planning techniques"[Title/Abstract] OR "planning techniques"[MeSH Terms] OR "health care facilities, manpower, and services"[MeSH Terms] OR "health care facility"[Title/Abstract] OR "health care facilities"[Title/Abstract] OR "administration"[Title/Abstract] OR "health administration"[Title/Abstract] OR "health care administration"[Title/Abstract] OR "healthcare administration"[Title/Abstract] OR "health services"[MeSH Terms] OR "health service"[Title/Abstract] OR "health practice"[Title/Abstract] OR "health administrator"[Title/Abstract] OR "health services administration"[Title/Abstract] OR "health services administration"[MeSH Terms] OR "health services needs and demand"[MeSH Terms] OR "health services needs and demand"[Title/Abstract] OR "health systems agencies"[MeSH Terms] OR "health visiting"[Title/Abstract] OR "healthcare service"[Title/Abstract] OR "medical health service"[Title/Abstract] OR "patient care planning"[Title/Abstract] OR "patient care planning"[MeSH Terms] OR "patient care plan"[Title/Abstract] OR "patient care management"[MeSH Terms] OR "patient care management"[Title/Abstract] OR "patient centered care"[Title/Abstract] OR "patient centered care"[MeSH Terms] OR "patient management"[Title/Abstract] OR "patient navigation"[Title/Abstract] OR "patient navigation"[MeSH Terms] OR “patient-centered care”[Title/Abstract] OR "interpersonal communication"[Title/Abstract] OR "medical communication"[Title/Abstract] OR "patient communication"[Title/Abstract] OR "transcription"[Title/Abstract] OR "financial management"[Title/Abstract] OR "financial management"[MeSH Terms]
#4	#1 AND #2 AND #3

### Screening process

For the process of selecting studies, the web-based collaborative software platform for literature reviews, Covidence (www.covidence.org), was used. All references found by the search strategies devised for the chosen bibliographic databases were imported into Covidence and duplicate references were automatically detected and removed. Covidence checks for duplicates both from within the set of references imported from the current database as well as against all previous imports from other databases. The screening of studies was carried out in a two-part process each by the same two independent reviewers, NLS and BB, with expertise in health care and computer science, respectively. The eligibility criteria were used by the reviewers in their assessments during both parts of the screening process but only recorded in Covidence as grounds for exclusion during the second part. The first part of the process was a title and abstract screening in which the reviewers read the titles and abstracts of all imported studies. Studies were automatically included if eligibility criteria were met in full according to the assessments of both reviewers while all studies in which the reviewers agreed in their assessments that they did not meet at least one eligibility criterion were automatically excluded. In the event of any conflicts between the reviewers’ assessments, a follow-up discussion period allowed for possible consensus to be reached in which case the given studies were included or excluded accordingly. If consensus could not be reached, the given studies were included for further screening during the second part of the process in order to minimize the early exclusion of potentially relevant studies. The second part of the process was a full-text screening of all included studies from the title and abstract screening that proceeded in the same way except, in the case of any conflicts in the reviewers’ assessments, consensus between the two reviewers was required to be reached during the follow-up discussion period and all studies that were subsequently deemed in-eligible must be excluded on the grounds of the same criterion (e.g., “not general practice” discussed below). All studies included upon completion of the full-text screening comprise the final set of studies reported in this scoping review.

### Data charting process

The process of charting the data extracted from the set of all included studies was completed with Covidence using a modified version of a standard data extraction template provided by Covidence for use in reviews. The same two reviewers who completed the process of selecting sources, independently charted the data from all included studies and resolved any conflicts with a follow-up discussion period in which consensus was mandatory.

#### Extracted data attributes

The modified data extraction template for the set of all included studies consisted of 14 data items corresponding to basic publication attributes and research-based attributes aimed at addressing the three topics emphasized in our stated objectives. The basic publication attributes extracted from all studies included author names, title, year of publication, country of origin, type of publication, and stated aim of study. The research-based attributes extracted from all studies concerned the following nine questions: (i) General practice – “What is the problem?”, “What data is used?”, and “How are GPs involved?”; (ii) Administrative task – “What is the task?”, “What needs improving?”, “How automated?”; and (iii) Machine learning – “What is the problem?”, “What methods are used?”, and “What evaluation measures?”. The results from the data charting process are presented in two tables with the basic publication attributes of all included studies provided in the first and a summary of the research-based attributes of these same studies provided in the second. For each table, the corresponding text summarizes the most important results.

## Results

**Fig. 1:**
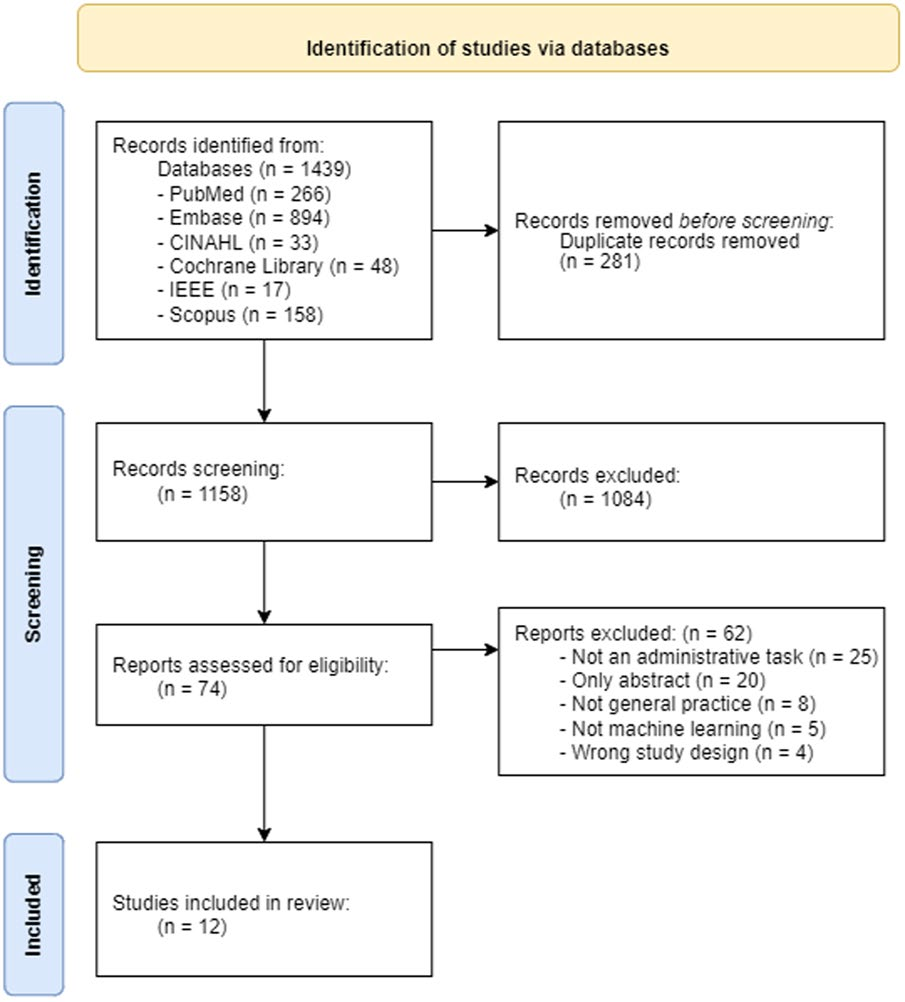
Process of identifying studies to include in the present scoping review

Figure 1 illustrates the data selection process: 1439 studies were identified in the six chosen databases of which 281 duplicates were removed, leaving 1158 studies for screening. Following the title and abstract screening process, 1084 studies were excluded, and 74 studies were included for further full-text screening. Of these 74 studies, a final total of 12 met all eligibility criteria needed to be included and collectively form the set of all studies reported in this scoping review.

### Basic publication attributes of included studies

Table 2 shows the six basic publication attributes of the complete set of 12 studies included following the completion of the identification and screening processes described above [15–26]. The years of publication in the included studies range from 1996 to 2022 with 10 studies published between 2017 and 2022 [15, 16, 18–24, 26]. Collectively, the studies cross eight countries (USA, Spain, Canada, Great Britain, Poland, Portugal, Australia, and New Zealand) with seven studies originating primarily from the USA [15, 16, 19–22, 26]. Eight of the studies are journal articles [15–18, 21, 22, 24, 26] and four studies are conference papers [19, 20, 23, 25]. The aims of study target various settings of general practice through a variety of problems, such as scheduling, classifying electronic health record text, care management, and facilitating interactions with electronic health records.

**Table 2:** Overview of the basic publication attributes of all included studies in the present scoping review

No	Author	Title	Year of publication	Country of origin	Type of publication	Aim of study
1	[15] Abu Lekham L., Wang Y., Hey E., Lam S. S., Khasawneh M. T	A Multi-Stage predictive model for missed appointments at outpatient primary care settings serving rural areas	2021	USA	Journal article	Prediction of missed appointments at outpatient primary care settings in rural areas using machine learning
2	[16] Ahmad M. U., Zhang A., Mhaskar R	A predictive model for decreasing clinical no-show rates in a primary care setting	2021	USA	Journal article	Development of a predictive model for patient no-shows or missed appointments in single physician family medicine practice
3	[17] Cubillas J. J., Ramos M. I., Feito F. R., Ureña T	An Improvement in the Appointment Scheduling in Primary Health Care Centers Using Data Mining	2014	Spain	Journal article	Creation of a model able to predict what kind of task (clinical, a medical certificate and issuing a prescription) patients daily require considering external factors influence
4	[18] López Seguí F., Ander Egg Aguilar R., de Maeztu G., García-Altés A., García Cuyàs F., Walsh S., et al	Teleconsultations between Patients and Healthcare Professionals in Primary Care in Catalonia: The Evaluation of Text Classification Algorithms Using Supervised Machine Learning	2020	Spain	Journal article	Evaluation of specific text classification algorithms for eConsulta messages and validate their predictive potential
5	[19] Michalowski, W., Michalowski, M., O'Sullivan, D., Wilk, S. and Carrier, M	AFGuide System to Support Personalized Management of Atrial Fibrillation	2017	Canada, Great Britain, Poland	Conference workshop technical report	Proposal of a clinical decision support system to educate and support primary care physicians in developing evidence-based and optimal atrial fibrillation therapies that consider multi-morbid conditions and patient preferences
6	[20] Mohammadi I., Mehrabi S., Sutton B., Wu H	Word Embedding and Clustering for Patient-Centered Redesign of Appointment Scheduling in Ambulatory Care Settings	2022	USA	Conference paper	Utilization of information from structured and unstructured electronic health records data to redesign appointment scheduling in community health clinics
7	[21] Mohammadi I., Wu H., Turkcan A., Toscos T., Doebbeling B. N	Data Analytics and Modeling for Appointment No-show in Community Health Centers	2018	USA	Journal article	Using predictive modeling techniques to develop and compare appointment no-show prediction models to better understand appointment adherence in underserved populations
8	[22] Park J., Kotzias D., Kuo P., Logan Iv R. L., Merced K., Singh S., et al	Detecting conversation topics in primary care office visits from transcripts of patient-provider interactions	2019	USA	Journal article	Investigation of the effectiveness of machine learning methods for automated annotation of medical topics in patient-provider dialog transcripts
9	[23] Peito, J. and Han, Q	Incorporating Domain Knowledge into Health Recommender Systems Using Hyperbolic Embeddings	2021	Portugal	Conference paper	Investigation of the possibility of a content-based recommender system for patient-doctor matchmaking by incorporating complex, domain-specific knowledge into the underlying model
10	[24] Schwartz J. L., Tseng E., Maruthur N. M., Rouhizadeh M	Identification of Prediabetes Discussions in Unstructured Clinical Documentation: Validation of a Natural Language Processing Algorithm	2022	USA	Journal article	Development and validation of a NLP pipeline to identify when providers discuss prediabetes management and treatment, which could later be used to determine if care delivered meets evidence-based guidelines and compare outcomes before and after an intervention
11	[25] Spenceley, S. E., Warren, J. R., Mudali, S. K. and Kirkwood, I. D	Intelligent Data Entry for Physicians by Machine Learning of an Anticipative Task Model	1996	Australia	Conference paper	Improve usability of electronic medical record systems by having the computer anticipate physicians’ data entry actions and generate short menus (hot lists) that offer likely selections to the user
12	[26] Williams A., Mekhail A., Williams J., McCord J., Buchan V	Effective resource management using machine learning in medicine: an applied example	2018	New Zealand, UK, USA	Journal article	Improve the efficiency of urgent lap sample processing using a transport scheduling platform applying machine learning techniques and simulate the efficiency and cost impact of the platform using historical data

### Research-based attributes of included studies

Table 3 summarizes the nine research-based attributes of the 12 included studies within the three topics of general practice, administrative task, and machine learning in fulfillment of our stated objectives.

**Table 3:** Summary of studies included in the present scoping review along nine research-based attributes concerning general practice, administrative tasks, and machine learning

	**Author**	**General practice**			**Administrative task**			**Machine learning**		
No		**What is the problem?**	**What data is used?**	**How are GPs** **involved?**	**What is the task?**	**What needs** **improving?**	**How** **automated?**	**What is the** **problem?**	**What methods are used?**	**What evaluation measures?**
1	[15] Abu Lekham et al. (2021)	Appointment scheduling	Data on patient appointments from an outpatient primary care center containing 26 features collected from 2016 to 2019	GPS not stated as involved in the research, but one author is affiliated with the healthcare center in question	Prediction of missed appointments (no-shows and early cancellations)	Patient scheduling, enhance capacity use, maximize revenues, minimize costs, and ultimately achieve financial stability	Fully	Supervised – binary, multi-class, multi-stage chain	Logistic Regression, Decision Tree, and some ensemble methods, including Random Forest, Ada Boost, Gradient Boosting, and Bagging	Precision, recall, F-value, and accuracy
2	[16] Ahmad et al. (2021)	Appointment scheduling	Patient-visit information (patient ID, month, day, age, gender, race, ethnicity, insurance type, visit type, and previous no-shows) from the EHR database, eClinicalWorks, between 2014 and 2016	GPs not stated as involved in the research, but all authors have medical affiliations	Reduce the rate of clinical no-shows or missed appointments	Decreasing clinical no-show rates	Fully	Supervised regression	Probit regression	Sensitivity, specificity, ROC curve and AUC
3	[17] Cubillas et al. (2014)	Appointment scheduling	Historical appointment data, weather and environmental for patients requiring administrative assistance during the years 2007, 2008, 2009, 2010, and 2011	GPs not stated as involved in the research and no authors have medical affiliations	Patient scheduling for differentiating between administrative and healthcare matters	Schedule in accordance with demand predicted for each day	Fully	Supervised regression	Generalized Linear Models and Support Vector Machines (with Linear and Gaussian kernel)	Average percentage error
4	[18] López Seguí et al. (2020)	Teleconsultation	Teleconsultations recorded by the teleconsulting system received between 2016 and 2018	GPs are involved in labelling the teleconsultations and some of the authors have medical affiliations	Text classification of teleconsultation messages between GPs and patients	Teleconsultation with decision support avoiding the need for a face-to-face visit	Fully	Supervised classification	Random Forest, Gradient Boosting (lightGBM), Fasttext, Multinomial Naive Bayes, and Naive Bayes Complement	Precision, sensitivity, F-value and the ROC curve
5	[19] Michalowski et al. (2017)	Care management	Canadian Cardiovascular Society’s atrial fibrillation clinical practice guidelines and Cochrane Database of Systematic Reviews	GPs not stated as involved in the research and no authors have medical affiliations	Disease management	Personalized management of atrial fibrillation	Fully	Supervised classification	Preference learning	No evaluation reported
6	[20] Mohammadi et al. (2022)	Appointment scheduling	EHR data (including patient, visit and provider characteristics) from encounters at an urban community health clinic in 2014 with an emphasis on the “schedulers’ notes” field	GPs not stated as involved in research, but authors are affiliated with health colleges and companies	Patient-centered re-design of appointment scheduling	Appointment scheduling based on patient needs	Partially	Unsupervised clustering	Agglomerative clustering	Clustering comparison with human judgements, scheduling assessments concerning average appointment duration, average time spent in clinic, number of patients seen by clinic
7	[21] Mohammadi et al. (2018)	Appointment scheduling	Semi-structured EHR data representing unique patients visiting a large urban multi-site community health center from 2014 to 2016	GPs not stated as involved in research, but authors are affiliated with health colleges and companies	Predict patients’ adherence to appointments	Appointment compliance and access to care	Fully	Supervised classification	Logistic regression, artificial neural network, and naïve Bayes classifier	AUC, sensitivity, positive (no-show) predictive value, overall accuracy
8	[22] Park et al. (2019)	Communication	Transcripts of audio recordings from primary care office visits at 26 ambulatory care clinics between 2007 and 2009	GPs not stated as involved in the research, but some of the authors have medical affiliations	Patient-provider communication	Patient satisfaction, payments, and quality of care	Fully	Supervised classification	Logistic classifiers, Support vector machines, Gated recurrent units, (Conditional random fields, Hidden Markov models, and hierarchical gated recurrent units)	Classification accuracy for talk-turns, precision, recall, F1 score
9	[23] Peito and Han (2021)	Healthcare recommender systems	Patients’ historical health records (with ICD-9 codes) from a European private health network	GPs not stated as involved in the research and no authors have medical affiliations	Patient-doctor matchmaking	Suggestions for patients concerning the best suited doctor for their next primary care visit	Partially	Representation learning (hyperbolic embeddings), transfer learning (pretrained embeddings and domain knowledge)	Domain knowledge filtering	Hit rate and precision
10	[24] Schwartz et al. (2022)	Care management	Prediabetes patients with an internal medicine primary care visit within an academic center with multiple ambulatory locations in Maryland and Washington, DC	GPs not stated as involved in the research, but all authors have medical affiliations	Physician–patient communication in pre-diabetes management	Guideline-concordant care	Fully	Supervised classification	Logistic regression, Linear support vector machines, Stochastic gradient descent, Random Forest, Decision tree, Gaussian naïve Bayes, Convolutional neural networks	Accuracy, sensitivity/recall, specificity, PPV/precision, F-measures
11	[25] Spenceley et al. (1996)	Electronic medical record (EMR) user interaction	SOAP ((S)ubjective complaint, (o)bjective findings, diagnosis or (a)nalysis, and therapy/ treatment (p)lan) notes for patients and visits from Adelaide General Practice	GPs not stated as involved in the research, but one author has a medical affiliation	Adaptive interface for data entry in EMR	Usability and support for data entry in EMRs	Fully	Supervised classification	Probabilistic	Hit rate
12	[26] Williams et al. (2019)	Resource management through scheduling	Private taxi contractor records of taxi journeys from November 2016–February 2017 and February 2017–June 2017	Interviews with primary care providers and all authors have medical backgrounds	Laboratory test scheduling	Time and cost reduction	Fully	Supervised regression	Linear regression	Time-to-result delay, cost reduction

#### General practice

The variety of current general practice problems identified concern appointment scheduling [15–17, 20, 21], teleconsultation [18], care management [19, 24], communication [22], healthcare recommender systems [23], user interaction with electronic medical records [25], and resource management through scheduling [26], with the most frequently occurring problem being appointment scheduling. Similarly, the data reportedly used in all studies differs both across all identified problems and within the same problem from different researchers, with sources largely consisting of proprietary data taken from a variety of domains, including actual general practice clinics [15, 17, 20–24], published clinical guidelines [19], electronic healthcare databases [16], and teleconsultation recordings [18], that differ considerably in their features. In looking at the level of involvement of GPs across all studies, it is not always clearly stated to what extent they participate in the actual research and only two studies clearly state involvement of GPs [18, 26]. Most of this involvement comes from the authors themselves as a majority have reported backgrounds affiliated in some way with medicine or health care.

#### Administrative task

As with the broad general practice problems identified, many of the specific administrative tasks concern scheduling [15–17, 20, 21] yet differ slightly within the given task of scheduling appointments e.g., predicting patients’ missed appointments (no-shows and early cancellations) [15], reducing the rate of clinical no-shows or missed appointments [16], and improved scheduling based on patient need [20]. Moreover, each of these appointment-scheduling tasks differs in the criteria they wish to improve, ranging from minimizing clinical costs or enhancing capacity to meeting daily demand or increasing access to care. Other administrative tasks identified concern teleconsultation support [18], disease management [19, 24], patient-provider communication [22], patient-doctor matchmaking [23], data entry in electronic health records [25], and laboratory test scheduling [26]. Importantly, all but two [20, 23] of the administrative tasks were identified as being fully automatable when assessing the technological contribution and reported workflow for addressing the given task.

#### Machine learning

In looking at the type of machine learning used, ten of the studies [15–19, 21, 22, 24–26] modelled their respective administrative tasks as supervised machine learning problems while one study [20] modelled its task as an unsupervised machine learning problem, and a final study [23] investigated the use of representation learning and transfer learning. The specific machine learning methods used to solve these problems varied widely from study to study with the single most frequently used technique being regression [15, 16, 21, 24, 26]. Roughly half of the studies include a “catch-all” approach in which the performances of several different machine learning methods are compared, however, few studies [21–24] employed modern data-driven methods of AI based on artificial neural networks. Despite the variety of different machine learning methods, most studies addressing a supervised machine learning problem employed traditional evaluation measures such as accuracy, precision, recall, or F-score [15, 18, 22–24] with some further opting to use measures more often found in the health sciences such as specificity [11, 13, 16, 19]. Notably, only one study employed evaluations with human judgements [20], one study elected to evaluate with respect to time and cost reductions [26], and one study employed no evaluation at all [19].

## Discussion

In this scoping review, we found that:

- ➔ Research regarding machine learning methods of AI applied to administrative tasks in general practice is either lacking or difficult to find when searching the databases primarily used in health care and the engineering sciences;
- ➔ The use and quantity of cutting-edge machine learning methods of AI applied to administrative tasks in general practice is significantly lower in comparison to what is found in diagnostic care;
- ➔ There is a wide variety of data used in terms of setting, type, and availability that makes it difficult to identify similar research questions and administrative tasks as well as compare the subsequent performance of the AI models developed; and
- ➔ It is difficult to determine the extent to which GPs were involved in the research and how needed such assistance is in administrative tasks.

### Strengths and limitations

The results suggest that research on machine learning methods for administrative tasks in general practice is either not widely pursued or possibly difficult for would-be researchers to find. In the former case, such a finding would simply mean that more research in this area is needed while the latter case would indicate that the chosen databases, search terms, and/or publication year range, used in this review were not appropriate. It is critical that researchers carrying out multi-disciplinary work, such as those interested in AI and administrative tasks in general practice, can easily find and build from previous related work. So, if typical administrative task keywords cannot be reliably used to find relevant publications in standard databases used within health care and the engineering sciences, more standardized keywords may need to be adopted or existing keywords should be better aligned with those used in each domain. While research on machine learning applied to administrative tasks in general practice appears limited, we believe the search strategies of this scoping review are sound. Nonetheless, it could be argued, for example, that the chosen search terms could be improved, as they do not align perfectly with the administrative tasks identified in [8]. However, using these exact terms in a preliminary search of the chosen databases resulted in few or no sources.

It could be further argued that, given the few numbers of studies, there were many that should have been included that were not, most likely due to the eligibility criterion concerning administrative tasks since the greatest number of studies (n = 25) were excluded on these grounds. However, these 25 excluded studies largely concerned either clearly diagnostic-related problems or national/regional statistics regarding quality improvement in primary care rather than solutions targeted at solving current problems in general practice clinics. Nonetheless, it is and may remain challenging to separate an administrative task from a diagnostic- or treatment-related task as general practice clinical duties frequently differ from one another, and primary care is constantly evolving. For example, while tasks concerning the direct treatment of disease would not be considered administrative tasks, there may be associated administrative tasks in one clinic involving disease management and treatment planning (e.g., generating plans for courses of treatment or explanations and visual overviews that inform patients in support of their recovery). Next, it should be noted that this review has by design possibly failed to acknowledge research on a variety of *non*-data-driven methods of AI applied to administrative tasks in general practice. For example, our identification constraints concerning a publication date of 1990 or later could well have excluded such research because methods such as knowledge-based and expert systems were widespread prior to this time and data-driven methods, such as artificial neural networks, were only beginning to appear. Finally, it could be that research on related but more general administrative tasks (e.g., efficient time scheduling or prioritization of employees) may have been recently carried out but was excluded on the grounds of not being general practice. Assuming such research would be relevant to general practice, this would indicate that it might be difficult to find and apply to this a new domain.

### Comparison with existing literature

Importantly, the findings from the present scoping review largely support the observation identified in an observational study [8] that administrative tasks are highly automatable and, combined with the observation in [12] that GPs are more likely to use AI systems that are oriented towards administrative-like tasks over diagnostic support systems, it appears necessary that further research in this area is needed. With respect to similar existing reviews on AI in general practice, [12, 27] have surveyed more general problems in general practice without a focus on administrative tasks. Consequently, the present scoping review provides a new, more focused point-of-view regarding the state of research concerning machine learning and administrative tasks in general practice. Finally, it is important to stress that this review has demonstrated that there is currently a significantly lower amount of research on machine learning applied to administrative tasks in general practice in comparison to the amount found in diagnostic care in general practice [12, 27].

### Implications for research and/or practice

This review has demonstrated that administrative tasks in general practice have relevant use cases suitable for academic research and high potential for being fully automated by data-driven methods of AI, yet the current quantity and use of cutting-edge machine learning methods (e.g., deep learning using artificial neural networks), when compared to those applied in diagnostic support, appear lacking. These issues are likely the result of a lack of available data and a general emphasis on diagnostic-based tasks over administrative ones. The reasoning for the latter issue is understandable, as the desire to directly improve care and minimize suffering is high. However, the necessity to reduce time-consuming administrative tasks required by practitioners can also go a long way in indirectly helping to improve care by reducing the workload of doctors so that they can focus their attention on tasks that demand more of their expertise. The former issue regarding the availability of relevant data needed for the successful deployment of the latest machine learning methods is perhaps more challenging to address. The sensitive and decentralized nature of patient medical information means that the data reported in the studies frequently varies in terms of setting (e.g., general practice clinic vs. community health care center), type (e.g., patient data vs. population statistics), and availability (e.g., existing open data collections vs. proprietary data resources). This makes it challenging for researchers to carry out replicable scientific research. Even though most of the studies addressed problems pertaining to scheduling, the data used from study to study varied and so the specific administrative tasks differed slightly. Consequently, the way in which to model these tasks as machine learning problems and which methods were employed, differed as well. This makes it difficult to compare performances of the systems directly despite many of the studies employing the same evaluation measures (e.g., precision and recall). Despite their variety, however, many of these tasks appear to be fully automatable.

Many of the sources do not make clear the extent to which GPs were involved in the research. In recent explainable artificial intelligence (XAI) efforts to solve various problems in the medical domain, for example, there is a growing need for doctors to be more involved in the development and evaluation of AI diagnostic support tools and systems [28]. It is not clear, however, whether this same need exists for administrative tasks in general practice, but the current level of GP involvement in administrative tasks appears low. It remains an open question whether medical professionals, that are not necessarily GPs, will be sufficient in addressing administrative tasks in general practice, as the potential level of involvement may vary from assistance in merely helping to identify the problem to annotating the data used or assisting in the development and evaluation of the AI system itself. There could be, for example, evaluation measures for administrative tasks based on time-reduction that require consideration of the GP or their expertise, such as scheduling patients according to cognitive load.

In summary, researchers would be well served going forward to avoid proprietary data sources that differ considerably in content from one another. This would ensure that research can be carried out on the same administrative tasks using the same machine learning paradigms that can be evaluated in the same way – leading to steadily improving models and research that can be replicated and cited by others. Researchers should also make it clearer the extent to which actual GPs were involved in the research, as this is a growing concern in XAI diagnostic support, and likely to be one for administrative tasks as well.

## Conclusions

In this scoping review, we provided a detailed look into the limited field of research developing in the application of machine learning to administrative tasks in general practice. The findings indicate that while there is a great need and high potential for using such methods, the current lack of a significant body of research is likely the result of an unavailability of open-source and standardized data sources as well as a general prioritization of diagnostic-related tasks over administrative ones. Future research would benefit from the use of open-source data, cutting-edge methods of machine learning, and clearly stated GP involvement.

## Supplementary Information

**Additional file 1.** Preferred Reporting Items for Systematic reviews and Meta-Analyses extension for Scoping Reviews (PRISMA-ScR) Checklist.

**Additional file 2.** Search strategy for Scoping Review.

## Data Availability

All data generated or analyzed during this study are included in this published article and its supplementary information files.

## Abbreviations

AI: Artificial Intelligence
GP: General Practitioner
XAI: Explainable Artificial Intelligence
