# Supplementary material for: Machine learning in general practice: scoping review of administrative task support and automation
Source: BMC Prim Care. 2023 Jan 14;24:14. doi: 10.1186/s12875-023-01969-y (PMC9840326; doi:10.1186/s12875-023-01969-y)
Supplement: Supplementary file 2 — Additional file 2. Search strategy for Scoping Review. [file 12875_2023_1969_MOESM2_ESM.pdf]

Search strategy for Scoping Review

Date: 04-20-2022

Eligibility criteria

**Population**	We included all studies concerning general practice	General practice, general medical practice, general medicine, primary medical care, primary health care, primary healthcare, health care primary, healthcare primary, primary care, family practice, family medicine, family medicine practice, private practice, first line care
**Intervention**	Machine learning (and paradigms within machine learning)	machine learning, supervised learning, unsupervised learning, reinforcement learning, semi-supervised learning, transfer learning, federated learning, deep learning
**Comparison**	No comparator	No comparator
**Outcomes**	Health care administration	Organization and administration, administration and planning, Administration and management, health information management, information management, management information systems, management, administrative health data, administration, planning techniques, health care facilities and services, health care facilities, manpower, and services, health facility, health care facilities, health facility administration, administrative personnel, health administration, health care administration, healthcare administration, health service*, health services administration, health services administration, health practice, health administrator, health systems agencies, health visiting, healthcare service, medical health service, health care management, patient care plan*, patient care management, patient(-)centered care, patient management, patient navigation, interpersonal communication, medical communication, patient communication, medical information, health communication, transcription, financial management

Search strategy

PubMed

No.	Search string	Results
#1	"general practice"[MeSH Terms] OR "general practice"[Title/Abstract] OR "general medicine"[Title/Abstract] OR "primary medical care"[Title/Abstract] OR "primary health care"[MeSH Terms] OR "primary health care"[Title/Abstract] OR "primary healthcare"[Title/Abstract] OR "health care primary"[Title/Abstract] OR "healthcare primary"[Title/Abstract] OR "primary care"[Title/Abstract] OR "family practice"[MeSH Terms] OR "family practice"[Title/Abstract] OR "family medicine"[Title/Abstract] OR "family medicine practice"[Title/Abstract] OR "private practice"[MeSH Terms] OR "private practice"[Title/Abstract] OR "first line care"[Title/Abstract]	375,511
#2	"machine learning"[MeSH Terms] OR "machine learning"[Title/Abstract] OR "learning machine"[Title/Abstract] OR "supervised machine learning"[MeSH Terms] OR "supervised learning"[Title/Abstract] OR "supervised machine learning"[Title/Abstract] OR "unsupervised machine learning"[MeSH Terms] OR "unsupervised machine learning"[Title/Abstract] OR "unsupervised learning"[Title/Abstract] OR "reinforcement learning"[Title/Abstract] OR "reinforcement machine learning"[Title/Abstract] OR "semi supervised machine learning"[Title/Abstract] OR "semi supervised learning"[Title/Abstract] OR "deep learning"[MeSH Terms] OR "deep learning"[Title/Abstract] OR "transfer learning"[Title/Abstract] OR "federated learning"[Title/Abstract]	103,793
#3	"administration and organization"[Title/Abstract] OR "administration and planning"[Title/Abstract] OR "management"[Title/Abstract] OR "management information systems"[MeSH Terms] OR "organization and administration"[MeSH Terms] OR "organization and administration"[Title/Abstract] OR "planning techniques"[Title/Abstract] OR "planning techniques"[MeSH Terms] OR "health care facilities, manpower, and services"[MeSH Terms] OR "health care facility"[Title/Abstract] OR "health care facilities"[Title/Abstract] OR "administration"[Title/Abstract] OR "health administration"[Title/Abstract] OR "health care administration"[Title/Abstract] OR "healthcare administration"[Title/Abstract] OR "health services"[MeSH Terms] OR "health service"[Title/Abstract] OR "health practice"[Title/Abstract] OR "health administrator"[Title/Abstract] OR "health services administration"[Title/Abstract] OR "health services administration"[MeSH Terms] OR "health services needs and demand"[MeSH Terms] OR "health services needs and demand"[Title/Abstract] OR "health systems agencies"[MeSH Terms] OR "health visiting"[Title/Abstract] OR "healthcare service"[Title/Abstract] OR "medical health service"[Title/Abstract] OR "patient care planning"[Title/Abstract] OR "patient care planning"[MeSH Terms] OR "patient care plan"[Title/Abstract] OR "patient care management"[MeSH Terms] OR "patient care management"[Title/Abstract] OR "patient	7,516,830

	centered care"[Title/Abstract] OR "patient centered care"[MeSH Terms] OR "patient management"[Title/Abstract] OR "patient navigation"[Title/Abstract] OR "patient navigation"[MeSH Terms] OR “patient-centered care”[Title/Abstract] OR "interpersonal communication"[Title/Abstract] OR "medical communication"[Title/Abstract] OR "patient communication"[Title/Abstract] OR "transcription"[Title/Abstract] OR "financial management"[Title/Abstract] OR "financial management"[MeSH Terms]	
#4	#1 AND #2 AND #3	426
#5	#4 AND Filters: Free full text, Full text, English, from 1990/1/1 - 2022/4/1	266

Embase

**No.**	**Search string**	**Results**
#1	'general practice'/exp OR 'family practice':ti,ab OR 'general medical practice':ti,ab OR 'general medicine':ti,ab OR 'general practice':ti,ab OR 'primary health care'/exp OR 'first line care':ti,ab OR 'health care, primary':ti,ab OR 'primary health care':ti,ab OR 'primary healthcare':ti,ab OR 'primary medical care'/exp OR 'medical care, primary':ti,ab OR 'primary care':ti,ab OR 'primary medical care':ti,ab OR 'family medicine'/exp OR 'family medicine':ti,ab OR 'private practice'/exp OR 'practice, private':ti,ab OR 'private practice':ti,ab	382,66
#2	'machine learning'/exp OR 'learning machine':ti,ab OR 'learning machines':ti,ab OR 'machine learning':ti,ab OR 'supervised machine learning'/exp OR 'supervised learning (machine learning)':ti,ab OR 'supervised machine learning':ti,ab OR 'unsupervised machine learning'/exp OR 'unsupervised learning (machine learning)':ti,ab OR 'unsupervised machine learning':ti,ab OR 'reinforcement learning (machine learning)'/exp OR 'reinforcement learning (machine learning)':ti,ab OR 'reinforcement learning algorithm':ti,ab OR 'reinforcement machine learning':ti,ab OR 'semi supervised machine learning'/exp OR 'semi supervised machine learning':ti,ab OR 'semi-supervised learning (machine learning)':ti,ab OR 'semisupervised learning (machine learning)':ti,ab OR 'semisupervised machine learning':ti,ab OR 'deep learning'/exp OR 'deep learning':ti,ab OR 'hierarchical learning':ti,ab OR 'transfer learning':ti,ab OR 'federated learning':ti,ab	318,257
#3	'organization and management'/exp OR 'organisation and administration':ti,ab OR 'organisation and management':ti,ab OR 'organization and administration':ti,ab OR 'organization and management':ti,ab OR 'ownership':ti,ab OR 'planning techniques':ti,ab OR 'privatization':ti,ab OR 'health care organization'/exp OR 'health care organisation':ti,ab OR 'health care organization':ti,ab OR 'health care rationing':ti,ab OR 'health care structure':ti,ab OR 'health organisation':ti,ab OR 'health organization':ti,ab OR 'health systems agencies':ti,ab OR 'healthcare organisation':ti,ab OR 'healthcare organization':ti,ab OR 'healthcare rationing':ti,ab OR 'healthcare structure':ti,ab OR 'organisation, health care':ti,ab OR 'organization,	10,364,354

	health care':ti,ab OR 'administration'/exp OR 'information system'/exp OR 'information management':ti,ab OR 'information system':ti,ab OR 'information systems':ti,ab OR 'integrated advanced information management systems':ti,ab OR 'management information system':ti,ab OR 'management information systems':ti,ab OR 'management, information':ti,ab OR 'personnel staffing and scheduling information systems':ti,ab OR 'medical information system'/exp OR 'clinical information system':ti,ab OR 'clinical pharmacy information systems':ti,ab OR 'health information exchange':ti,ab OR 'health information management':ti,ab OR 'health information manager':ti,ab OR 'health information network':ti,ab OR 'health information system':ti,ab OR 'health information systems':ti,ab OR 'medical information service':ti,ab OR 'medical information system':ti,ab OR 'physician-practice-management information system':ti,ab OR 'management'/exp OR 'institutional management teams':ti,ab OR 'management':ti,ab OR 'management audit':ti,ab OR 'office management':ti,ab OR 'pharmacy administration':ti,ab OR 'practice management':ti,ab OR 'practice management, medical':ti,ab OR 'practice valuation and purchase':ti,ab OR 'administrative personnel'/exp OR 'administrative personnel':ti,ab OR 'administrative staff':ti,ab OR 'administrative worker':ti,ab OR 'administrator':ti,ab OR 'personnel, administrative':ti,ab OR 'administrative health data'/exp OR 'administrative data (health)':ti,ab OR 'administrative health care data':ti,ab OR 'administrative health data':ti,ab OR 'administrative healthcare data':ti,ab OR 'health administrative data':ti,ab OR 'health care administrative data':ti,ab OR 'healthcare administrative data':ti,ab OR 'health care facilities and services'/exp OR 'health care facilities and services':ti,ab OR 'health care facilities, manpower, and services':ti,ab OR 'health care facility'/exp OR 'health care facility':ti,ab OR 'health facilities':ti,ab OR 'health facilities, proprietary':ti,ab OR 'health facility':ti,ab OR 'health facility administrator':ti,ab OR 'health facility administrators':ti,ab OR 'health facility closure':ti,ab OR 'health facility environment':ti,ab OR 'health facility merger':ti,ab OR 'health facility moving':ti,ab OR 'health facility planning':ti,ab OR 'health facility size':ti,ab OR 'healthcare facility':ti,ab OR 'medical office buildings':ti,ab OR 'patients` rooms':ti,ab OR 'physicians` offices':ti,ab OR 'health care management'/exp OR 'annual reports':ti,ab OR 'annual reports as topic':ti,ab OR 'health administration':ti,ab OR 'health administrator':ti,ab OR 'health care administration':ti,ab OR 'health care management':ti,ab OR 'health management':ti,ab OR 'healthcare administration':ti,ab OR 'healthcare management':ti,ab OR 'health service'/exp OR 'health care agency':ti,ab OR 'health care service':ti,ab OR 'health practice':ti,ab OR 'health service':ti,ab OR 'health services':ti,ab OR 'health services administration':ti,ab OR 'health system agency':ti,ab OR 'health visiting':ti,ab OR 'healthcare agency':ti,ab OR 'healthcare service':ti,ab OR 'medical health service':ti,ab OR 'personal health services':ti,ab OR 'physician service':ti,ab OR 'service, health':ti,ab OR 'health systems agency'/exp OR 'patient care planning'/exp OR 'patient care plan':ti,ab OR 'patient care planning':ti,ab OR 'patient	

	care'/exp OR 'care, continuity of':ti,ab OR 'continuity of care':ti,ab OR 'continuity of patient care':ti,ab OR 'episode of care':ti,ab OR 'patient care':ti,ab OR 'patient care management':ti,ab OR 'patient care team':ti,ab OR 'patient centered care':ti,ab OR 'patient helper':ti,ab OR 'patient management':ti,ab OR 'patient navigation':ti,ab OR 'patient-centered care':ti,ab OR 'patient centered communication'/exp OR 'interpersonal communication'/exp OR 'communication':ti,ab OR 'communication (interpersonal)':ti,ab OR 'interpersonal communication':ti,ab OR 'medical information'/exp OR 'health communication':ti,ab OR 'health information':ti,ab OR 'information, medical':ti,ab OR 'medical information':ti,ab OR 'transcription'/exp OR 'financial management'/exp OR 'financial management':ti,ab OR 'financial support':ti,ab OR 'financing':ti,ab OR 'financing, construction':ti,ab OR 'financing, organised':ti,ab OR 'management, financial':ti,ab OR 'financing, organized':ti,ab	
#4	#1 AND #2 AND #3	983
#5	#4 AND ([article]/lim OR [article in press]/lim OR [conference abstract]/lim OR [conference paper]/lim OR [conference review]/lim OR [review]/lim) AND [english]/lim AND [1990-2022]/py	894

CINAHL

**No.**	**Search string**	**Results**
#1	MJ "general practice" OR AB "general practice" OR MJ "family practice" OR AB "family practice" OR MJ "general medical practice" OR AB "general medical practice" OR MJ "general medicine" OR AB "general medicine" OR MJ "primary medical care" OR AB "primary medical care" OR MJ "primary care" OR AB "primary care" OR MJ "primary health care" OR AB "primary health care" OR MJ "first line care" OR AB "first line care" OR MJ "primary healthcare" OR AB "primary healthcare" OR MJ "private practice" OR AB "private practice"	132,461
#2	MJ "machine learning" OR AB "machine learning" OR MJ "learning machine" OR AB "learning machine" OR MJ "supervised machine learning" OR AB "supervised machine learning" OR MJ "supervised learning" OR AB "supervised learning" OR MJ "unsupervised machine learning" OR AB "unsupervised machine learning" OR MJ "unsupervised learning" OR AB "unsupervised learning" OR MJ "reinforcement learning" OR AB "reinforcement learning" OR MJ "reinforcement learning algorithm" OR AB "reinforcement learning algorithm" OR MJ "reinforcement learning algorithms" OR AB "reinforcement learning algorithms" OR MJ "semi supervised machine learning" OR AB "semi supervised machine learning" OR MJ "semi supervised machine learning algorithm" OR AB "semi supervised machine learning algorithm" OR MJ "semisupervised machine learning" OR AB "semisupervised machine learning" OR MJ "semisupervised machine learning methods" OR AB "semisupervised machine learning methods" OR MJ "deep learning" OR AB "deep learning" OR MJ "transfer learning" OR AB "transfer learning" OR MJ "federated learning" OR AB "federated learning"	10,892
#3	MJ "administration and organization" OR AB "administration and organization" OR MJ "administration and planning" OR AB "administration and planning" OR	1,003,678

	MJ "management" OR AB "management" OR MJ "management information systems" OR AB "management information systems" OR MJ "organization and administration" OR AB "organization and administration" OR AB "organization and administration" OR MJ "planning techniques" OR AB "planning techniques" OR MJ "health care facilities, manpower, and services" OR AB "health care facilities, manpower, and services" OR MJ "health care facility" OR AB "health care facility" OR MJ "health care facilities" OR AB "health care facilities" OR MJ "administration" OR AB "administration" OR MJ "health administration" OR AB "health administration" OR MJ "health care administration" OR AB "health care administration" OR MJ "healthcare administration" OR AB "healthcare administration" OR MJ "health services" OR AB "health services" OR MJ "health service" OR AB "health service" OR MJ "health practice" OR AB "health practice" OR MJ "health administrator" OR AB "health administrator" OR MJ "health services administration" OR AB "health services administration" OR MJ "health services needs and demand" OR AB "health services needs and demand" OR MJ "health systems agencies" OR AB "health systems agencies" OR MJ "health visiting" OR AB "health visiting" OR MJ "healthcare service" OR AB "healthcare service" OR MJ "medical health service" OR AB "medical health service" OR MJ "patient care planning" OR AB "patient care planning" OR MJ "patient care plan" OR AB "patient care plan" OR MJ "patient care management" OR AB "patient care management" OR MJ "patient centered care" OR MJ "patient centered care" OR MJ "patient management" OR AB "patient management" OR MJ "patient navigation" OR AB "patient navigation" OR MJ "patient-centered care" OR AB "patient-centered care" OR MJ "interpersonal communication" OR AB "interpersonal communication" OR MJ "medical communication" OR AB "medical communication" OR MJ "patient communication" OR AB "patient communication" OR MJ "transcription" OR AB "transcription" OR MJ "financial management" OR AB "financial management"	
#4	#1 AND #2 AND #3	34
#5	Limiters - Published Date: 19900101-20220431; English Language	33

Cochrane Library

No.	Search string	Results
#1	MeSH descriptor: [General Practice] explode all trees	2497
#2	"general practice"	7501
#3	MeSH descriptor: [Family Practice] explode all trees	1980
#4	"family practice"	4242
#5	"general medical practice"	63
#6	"general medicine"	1102
#7	"primary medical care"	5910
#8	"primary care"	23678
#9	MeSH descriptor: [Primary Health Care] explode all trees	8252
#10	"primary health care"	8023
#11	"first line care"	22
#12	"primary healthcare"	948
#13	MeSH descriptor: [Private Practice] explode all trees	114

#14	"private practice"	1058
#15	#1 OR #2 OR #3 OR #4 OR #5 OR #6 OR #7 OR #8 OR #9 OR #10 OR #11 OR #12 OR #13 OR #14	38,809
#16	MeSH descriptor: [Machine Learning] explode all trees	206
#17	"machine learning"	1833
#18	"learning machine"	53
#19	MeSH descriptor: [Supervised Machine Learning] explode all trees	24
#20	"supervised machine learning"	89
#21	"supervised learning"	38
#22	MeSH descriptor: [Unsupervised Machine Learning] explode all trees	0
#23	"unsupervised machine learning"	26
#24	"unsupervised learning"	14
#25	"reinforcement learning"	163
#26	"reinforcement learning algorithm"	3
#27	"reinforcement learning algorithms"	2
#28	"semi supervised machine learning"	2
#29	"semi supervised machine learning algorithm"	0
#30	"semisupervised machine learning"	2
#31	"semisupervised machine learning methods"	1
#32	MeSH descriptor: [Deep Learning] explode all trees	43
#33	"deep learning"	697
#34	"transfer learning"	67
#35	"federated learning"	2
#36	#26 OR #27 OR #28 OR #29 OR #30 OR #31 OR #32 OR #33 OR #34 OR #35 OR #36 OR #37 OR #38 OR #39 OR #40 OR #41	2,643
#37	MeSH descriptor: [Organization and Administration] explode all trees	39,456
#38	"administration and organization"	1
#39	"administration and planning"	1
#40	MeSH descriptor: [Health Information Management] explode all trees	14
#41	MeSH descriptor: [Information Management] explode all trees	3,729
#42	"management"	146,182
#43	"information management"	191
#44	"management information systems"	30
#45	"organization and administration"	56
#46	MeSH descriptor: [Planning Techniques] explode all trees	36
#47	"planning techniques"	96
#48	"health care facilities, manpower, and services"	3
#49	MeSH descriptor: [Health Care Facilities, Manpower, and Services] explode all trees	114,774
#50	MeSH descriptor: [Health Facilities] explode all trees	16,055
#51	"Health care facilities"	301
#52	MeSH descriptor: [Administrative Personnel] explode all trees	110
#53	"administration"	368,048
#54	"health care administration"	93
#55	"healthcare administration"	22
#56	MeSH descriptor: [Health Services] explode all trees	103,582
#57	"health services"	17,870
#58	MeSH descriptor: [Health Services Administration] explode all trees	205,914

#59	"health practice"	329
#60	"health administrator"	3
#61	"health services administration"	106
#62	MeSH descriptor: [Health Services Needs and Demand] explode all trees	491
#63	"health services needs and demands"	0
#64	MeSH descriptor: [Health Systems Agencies] explode all trees	3
#65	"health visiting"	42
#66	"healthcare service"	224
#67	"medical health service"	3
#68	MeSH descriptor: [Patient Care Planning] explode all trees	1,801
#69	"patient care planning"	750
#70	"patient care plan"	23
#71	"patient care management"	198
#72	MeSH descriptor: [Patient Care Management] explode all trees	27,171
#73	MeSH descriptor: [Patient-Centered Care] explode all trees	832
#74	"patient centered care"	1,232
#75	"Patient-Centered Care"	1,232
#76	"patient management"	1,331
#77	"patient navigation"	457
#78	MeSH descriptor: [Patient Navigation] explode all trees	149
#79	"interpersonal communication"	2,307
#80	"medical communication"	65
#81	"patient communication"	512
#82	MeSH descriptor: [Health Communication] explode all trees	272
#83	"transcription"	6,208
#84	MeSH descriptor: [Financial Management] explode all trees	268
#85	"financial management"	724
#86	#43 OR #44 OR #45 OR #46 OR #47 OR #48 OR #49 OR #50 OR #51 OR #52 OR #53 OR #54 OR #55 OR #56 OR #57 OR #58 OR #59 OR #60 OR #61 OR #62 OR #63 OR #64 OR #65 OR #66 OR #67 OR #68 OR #69 OR #70 OR #71 OR #72 OR #73 OR #74 OR #75 OR #76 OR #77 OR #78 OR #79 OR #80 OR #81 OR #82 OR #83 OR #84 OR #85 OR #86 OR #87 OR #88 OR #89 OR #90 OR #91	644,079
#87	#25 AND #42 AND #92	73
#88	with Cochrane Library publication date from Jan 1990 to Apr 2022, in Cochrane Reviews and Cochrane Protocols	48

<u>IEEE</u>

No.	Search string	Results
#1	("All Metadata":"general practice") OR ("All Metadata":"general medical practice") OR ("All Metadata":"general medicine") OR ("All Metadata":"general medical care") OR ("All Metadata":"primary health care") OR ("All Metadata":"primary healthcare") OR ("All Metadata":"health care primary") OR ("All Metadata":"healthcare primary") OR ("All Metadata":"primary care") OR ("All Metadata":"family practice") OR ("All Metadata":"family medicine") OR ("All Metadata":"family medicine practice") OR ("All Metadata":"private practice") OR ("All Metadata":"first line care")	962

#2	("All Metadata":"machine learning") OR ("All Metadata":"learning machine*") OR ("All Metadata":"supervised machine learning") OR ("All Metadata":"supervised learning") OR ("All Metadata":"unsupervised machine learning") OR ("All Metadata":"unsupervised learning") OR ("All Metadata":"reinforcement learning") OR ("All Metadata":"reinforcement machine learning") OR ("All Metadata":"semi supervised machine learning") OR ("All Metadata":"semi supervised learning") OR ("All Metadata":"deep learning") OR ("All Metadata":"transfer learning") OR ("All Metadata":"federated learning")	215,923
#3	("All Metadata":"administration and organization") OR ("All Metadata":"administration and planning") OR ("All Metadata":"management") OR ("All Metadata":"management information systems") OR ("All Metadata":"organization and administration") OR ("All Metadata":"planning techniques") OR ("All Metadata":"health care facilities, manpower, and services") OR ("All Metadata":"health care facility*") OR ("All Metadata":"administration") OR ("All Metadata":"health administration") OR ("All Metadata":"health care administration") OR ("All Metadata":"healthcare administration") OR ("All Metadata":"health service*") OR ("All Metadata":"health administrator") OR ("All Metadata":"health services administration") OR ("All Metadata":"health services needs and demand") OR ("All Metadata":"health systems agencies") OR ("All Metadata":"health visiting") OR ("All Metadata":"healthcare service") OR ("All Metadata":"medical health service") OR ("All Metadata":"patient care plan*") OR ("All Metadata":"patient care management") OR ("All Metadata":"patient centered care") OR ("All Metadata":"patient management") OR ("All Metadata":"patient navigation") OR ("All Metadata":"patient-centered care") OR ("All Metadata":"interpersonal communication") OR ("All Metadata":"medical communication") OR ("All Metadata":"patient communication") OR ("All Metadata":"transcription") OR ("All Metadata":"financial management")	622,164
#4	#1 AND #2 AND #3	17

Scopus

No.	Search string	Results
#1	(TITLE-ABS-KEY ("general practice") OR TITLE-ABS-KEY ("general medical practice") OR TITLE-ABS-KEY ("general medicine") OR TITLE-ABS-KEY ("general medical care") OR TITLE-ABS-KEY ("primary health care") OR TITLE-ABS-KEY ("primary healthcare") OR TITLE-ABS-KEY ("health care primary") OR TITLE-ABS-KEY ("healthcare primary") OR TITLE-ABS-KEY ("primary care") OR TITLE-ABS-KEY ("family practice") OR TITLE-ABS-KEY ("family medicine") OR TITLE-ABS-KEY ("family medicine practice") OR TITLE-ABS-KEY ("private practice") OR TITLE-ABS-KEY ("first line care"))	357,436
#2	(TITLE-ABS-KEY ("machine learning") OR TITLE-ABS-KEY ("learning machine") OR TITLE-ABS-KEY ("supervised machine learning") OR TITLE-ABS-KEY ("supervised learning") OR TITLE-ABS-KEY ("unsupervised machine learning") OR TITLE-ABS-KEY ("unsupervised learning") OR TITLE-ABS-KEY ("reinforcement machine learning") OR	588,958

	TITLE-ABS-KEY ("reinforcement learning") OR TITLE-ABS-KEY ("semi supervised machine learning") OR TITLE-ABS-KEY ("semi supervised learning") OR TITLE-ABS-KEY ("deep learning") OR TITLE-ABS-KEY ("transfer learning") OR TITLE-ABS-KEY ("federated learning"))	
#3	(TITLE-ABS-KEY ("administration and organization") OR TITLE-ABS-KEY ("administration and planning") OR TITLE-ABS-KEY ("management") OR TITLE-ABS-KEY ("management information systems") OR TITLE-ABS-KEY ("organization and administration") OR TITLE-ABS-KEY ("planning techniques") OR TITLE-ABS-KEY ("health care facilities, manpower, and services") OR TITLE-ABS-KEY ("health care facility") OR TITLE-ABS-KEY ("administration") OR TITLE-ABS-KEY ("health administration") OR TITLE-ABS-KEY ("health care administration") OR TITLE-ABS-KEY ("healthcare administration") OR TITLE-ABS-KEY ("health service*") OR TITLE-ABS-KEY ("health administrator") OR TITLE-ABS-KEY ("health services administration") OR TITLE-ABS-KEY ("health services needs and demands") OR TITLE-ABS-KEY ("health systems agencies") OR TITLE-ABS-KEY ("health visiting") OR TITLE-ABS-KEY ("healthcare service") OR TITLE-ABS-KEY ("medical health service") OR TITLE-ABS-KEY ("patient care plan*") OR TITLE-ABS-KEY ("patient care management") OR TITLE-ABS-KEY ("patient centered care") OR TITLE-ABS-KEY ("patient-centered care") OR TITLE-ABS-KEY ("patient management") OR TITLE-ABS-KEY ("patient navigation") OR TITLE-ABS-KEY ("interpersonal communication") OR TITLE-ABS-KEY ("medical communication") OR TITLE-ABS-KEY ("patient communication") OR TITLE-ABS-KEY ("transcription") OR TITLE-ABS-KEY ("financial management"))	8,897,824
#4	#1 AND #2 AND #3 AND (LIMIT-TO (DOCTYPE , "ar") OR LIMIT-TO (DOCTYPE , "cp") OR LIMIT-TO (DOCTYPE , "re") OR LIMIT-TO (DOCTYPE , "cr")) AND (LIMIT-TO (LANGUAGE , "English"))	158
